# Turning Constraints into Adaptive Behavior: Secondary Pre-Service Teachers’ Bricolage and Agency in Physical Education

**DOI:** 10.3390/bs16040515

**Published:** 2026-03-29

**Authors:** Hyeyoun Park

**Affiliations:** Department of Physical Education, Institute of Sport Science, Seoul National University, Seoul 08826, Republic of Korea; ggun82@snu.ac.kr

**Keywords:** Safe Deficit, adaptive bricolage, teacher agency, behavioral adaptation, adaptive expertise, professional resilience, physical education, secondary education, teacher professional development

## Abstract

As secondary educational environments face increasing volatility due to systemic resource constraints and pedagogical uncertainty, understanding the behavioral mechanisms of teacher agency has become paramount. While traditional teacher education has emphasized the execution of standardized curricula, the current era demands a fundamental shift toward adaptive expertise and psychological resilience. This study investigates the processes by which 28 secondary pre-service physical education teachers (PSTs) navigate instructional resource deficits through the lens of adaptive behavior (bricolage) and ecological teacher agency. Utilizing a qualitative case study design, I collected data from two universities in Seoul, South Korea, through reflective journals, revised lesson plans, and micro-teaching video analysis reports over a full 15-week semester. The results identified five coordinates of an adaptive instructional design compass: (1) Facing Constraints, (2) Resource Mining, (3) Contextual Engineering, (4) Simulation, and (5) Reflective Participation. These coordinates represent a transformative behavioral process where PSTs convert environmental deficits into professional assets. The findings reveal distinct adaptation styles based on psychological dispositions: the analytically oriented group (Group A) prioritized structural redesign through digital tools, while the narratively oriented group (Group B) utilized human-centric somatic metaphors and virtual rehearsals to bridge the epistemic void. Crucially, this research suggests that teacher adaptation is not a mere technical adjustment but a dynamic behavioral achievement of agency that ensures the long-term instructional quality of physical education. I propose that teacher education programs should incorporate “Safe Deficit” simulations—carefully calibrated instructional constraints—to trigger adaptive behavior and ensure that future educators can thrive in unpredictable pedagogical contexts without the risk of professional burnout.

## 1. Introduction

In an era characterized by global uncertainty and rapid environmental shifts, the behavioral adaptation and psychological resilience required for instructional continuity in physical education (PE) face unprecedented challenges (Kirk, 2010). As educational environments become increasingly volatile, the traditional reliance on standardized resources is being questioned, necessitating a fundamental shift in how we perceive the psychological mechanisms of teacher agency. One of the core missions of Physical Education Teacher Education (PETE) is to empower pre-service teachers (PSTs), specifically junior-level students in their final academic year before their formal teaching practicum, to bridge the gap between university-taught theories and the unpredictable, often resource-depleted complexities of actual school environments.

Traditionally, instructional design has been grounded in technical rationality, a linear-rational process derived from Tyler’s curriculum theory (Tyler, 2013). This paradigm instills in PSTs a persistent myth: that instructional design is a precise map created through a perfect analysis of national standards and textbooks. The expectation is that if the map is drawn correctly, the classroom can be fully controlled and managed without error (Siedentop & Tannehill, 2000; Tinning, 2010). However, this study critiques such linear pedagogical models, arguing that they fail to account for the non-linear and messy reality of actual pedagogical practice, where instructional success depends more on situational improvisation than on the rigid execution of a pre-set script.

In the context of PE, this transition is particularly taxing due to the unique environmental and spatial constraints inherent to the subject, such as unpredictable weather, gym availability, and the fluctuating nature of student movement in open spaces. For secondary PSTs, the actual pedagogical field often betrays these idealistic maps. As Schön (2017) poignantly observed, practitioners do not operate in a stable laboratory but in the “swampy lowlands”—a realm of messiness where rigid plans are rendered ineffective. When PSTs encounter this harsh reality of the field, it often manifests as a significant instructional transition challenge, triggering a psychological discrepancy between university ideal pedagogy and actual school-based practice (Veenman, 1984; Kelchtermans & Ballet, 2002; O’Sullivan, 2006). This phenomenon necessitates a re-examination of teacher agency not as an innate, fixed capacity, but as a dynamic behavioral achievement emerging within restrictive environments (Biesta et al., 2015).

Recent scholarship emphasizes that professional development must move beyond simple knowledge transmission and foster adaptive behavioral methodologies (UNESCO, 2017). Beltman et al. (2011) further argue that the emotional and adaptive resilience of future educators is a key determinant for long-term quality, particularly when facing environmental and social constraints. Consequently, this study is primarily situated within the Ecological Perspective of Teacher Agency (Priestley et al., 2015; Patton, 2002), which posits that agency is not an innate property of the individual but is achieved through the dynamic interplay between a teacher’s individual psychological dispositions and the specific cultural and structural affordances of their environment.

In the micro-teaching context of this study, PSTs encountered two debilitating deficits that threatened the continuity of instruction. The first is the Epistemic Void, defined here as the gap where abstract textbook content fails to provide concrete, field-ready guidance. The second is the Physical Blockade, involving equipment shortages or spatial constraints that neutralize even the meticulously prepared lesson plans (Penney, 1998). While conventional research often adopts a deficit view—attributing these struggles to a lack of preparation or reflective thinking—this study reinterprets these gaps as ecological catalysts for teacher agency. I argue that these deficits are not obstacles to be eliminated but are “fertile voids” that facilitate the manifestation of latent professional vision and creative problem-solving.

Drawing on Lévi-Strauss (1962)’s distinction between the engineer and the bricoleur, this study reconceptualizes the PST as an adaptive bricoleur from a behavioral science perspective. Bricolage is not mere tinkering; it is a sophisticated cognitive and behavioral reorganization of reassembling digital tools, social networks, and somatic metaphors to maintain learning continuity. Teacher agency, in this sense, is not a fixed trait but an ecological achievement resulting from the behavioral interaction between the teacher’s disposition and their environment (Priestley et al., 2015; Patton, 2002). Specifically, the Safe Deficit paradigm—defined here as a managed and supportive instructional vacuum designed to prevent overwhelming instructional transition challenges—is proposed as a psychological scaffold for fostering adaptive expertise. Crucially, this paradigm does not discard theoretical pedagogical maps; rather, it utilizes them as essential foundational tools to transform PSTs from technical executors into resilient pedagogical architects who navigate uncertainty with an adaptive professional compass.

Despite the potential of bricolage as a sustainable pedagogical strategy, empirical research on the specific mechanisms through which PSTs achieve agency through bricolage in PE contexts is limited. In particular, understanding how different psychological dispositions—analytical versus narrative—influence these adaptive behaviors is crucial for developing personalized and resilient teacher education programs. This research contributes to the discourse on teacher professionalism by illustrating how ecological agency is achieved as a behavioral response to environmental constraints.

Therefore, this study aims to investigate the process by which secondary PSTs convert environmental deficits into professional assets. Specifically, the present study addresses the following questions: (1) What are the behavioral mechanisms of adaptive bricolage employed by PSTs when facing instructional constraints? (2) How do individual psychological dispositions differentiate these strategies? (3) How does the achievement of teacher agency manifest as a behavioral response to the Safe Deficit within a PE ecology?

## 2. Materials and Methods

### 2.1. Research Design: Qualitative Case Study

This study utilized a qualitative case study design to explore the adaptive mechanisms of pre-service teachers (PSTs) in-depth. According to Tisdell et al. (2025) and Stake (1995), a case study is uniquely suited for holistically understanding complex social phenomena within their real-world contexts. Rather than isolating variables in a controlled environment, this approach focuses on the messiness of instructional design as it unfolds within the swampy lowlands of actual pedagogical practice. By employing a case study approach, I was able to capture the temporal evolution of teacher agency—from the initial instructional transition challenge of curriculum failure (the gap between intended goals and situational execution) to the creative achievement of bricolage. The study interpreted the lived experiences and artifacts of PSTs through the theoretical lenses of ecological teacher agency and behavioral bricolage.

### 2.2. Participants and Context

The participants were 28 junior-level secondary PSTs (18 from University A and 10 from University B) majoring in Physical Education at two prominent universities in Seoul, South Korea. The sample size of 28 was determined based on the principle of data saturation in qualitative inquiry (Patton, 2002), where the continuous collection of reflective journals and video reports reached a point where no new thematic categories or theoretical insights regarding bricolage mechanisms were emergent. To ensure data richness and the credibility of the findings, the study employed purposive sampling. The selection criteria were: (1) enrollment in the major requirement course Research in PE Teaching Materials and Methods; (2) direct experience of pedagogical constraints due to epistemic or physical deficits during micro-teaching; and (3) active engagement in adaptive strategies to sustain instruction. This specific context—a capstone course focused on pedagogical content knowledge (PCK)—provided a natural laboratory to observe how upcoming educators negotiate the gap between theory and practice.

To explore how individual psychological dispositions interact with environmental constraints, participants were categorized into two distinct groups. It is crucial to emphasize that this categorization was not a pre-determined experimental condition but an emergent finding derived from the bottom-up inductive analysis of the 15-week empirical data of the PSTs’ behavioral responses and reflective patterns:

- Group A (Analytical/Log-centric, *n* = 18): PSTs who exhibited systematic behavioral responses through logical resource procurement and structural redesign.
- Group B (Narrative/Somatic, *n* = 10): PSTs who exhibited intuitive behavioral responses using metaphors and cultural codes.

### 2.3. Ethical Considerations

The study was conducted in accordance with the ethical guidelines of the institutional review board. Formal approval was waived because the research analyzed anonymized pedagogical artifacts produced within the standard curriculum, posing no more than minimal risk to participants. All procedures were conducted in accordance with the Declaration of Helsinki. Informed consent for the research use of these materials was obtained from all 28 participants, and any potentially identifiable information was removed prior to analysis.

### 2.4. Data Collections and Triangulation

To ensure the credibility and confirmability of the findings, data collection spanned a full 15-week semester. To achieve methodological rigor, multiple data sources were employed to facilitate triangulation (Patton, 2002), ensuring that the findings were not dependent on a single perspective. The three primary data sources were utilized for data triangulation, ensuring a multidimensional analysis of PSTs’ behaviors and reflections:

- Reflective Journals: Captured PSTs’ internal conflicts and psychological decision-making processes immediately after instructional design and micro-teaching sessions, documenting both reflection-in-action and reflection-on-action (Schön, 2017).
- Lesson Plans: The research tracked the behavioral transition of instructional design by comparing the initial (pre-revision) Map with the final (field-adjusted) Compass modified after encountering field constraints. This allowed for the identification of specific structural changes in pedagogical logic.
- Micro-teaching Video Analysis Reports: PSTs monitored their own performance videos to identify non-verbal behavioral interactions, physical habits, and improvised coping strategies. These reports provided a somatic bridge between the PSTs’ intended actions and their actual embodied behavioral performances.

### 2.5. Data Analysis: Inductive Content Analysis and Constant Comparison

Data organization and initial coding were facilitated using NVivo 12 (QSR International, Melbourne, Australia), and supplementary data management was performed using Microsoft Excel 2021 (Microsoft Corp., Redmond, WA, USA) to ensure systematic analysis. Data analysis followed the six-phase thematic analysis framework proposed by Braun and Clarke (Braun & Clarke, 2006): (1) familiarizing with the data, (2) generating initial codes, (3) searching for themes, (4) reviewing potential themes, (5) defining and naming themes, and (6) producing the report. All collected data were analyzed using inductive content analysis to identify recurring behavioral patterns and latent psychological themes related to teacher agency.

To ensure the rigor of the analytical process, a hierarchical coding scheme was developed. This involved moving from open codes (e.g., “re-purposing digital clips”) to broader sub-categories (e.g., “resource mining”), which were eventually synthesized into the overarching “Five Coordinates of an Adaptive Instructional Design Compass.” This systematic progression ensured that the final themes were firmly grounded in the raw empirical data rather than the researchers’ pre-conceived notions. Furthermore, to ensure that the identified bricolage mechanisms were not institutional artifacts but universal adaptation strategies, the Constant Comparative Method was employed between Group A and Group B throughout the analysis process.

Independent coding was conducted by the researcher with the assistance of an external peer reviewer to minimize individual bias. To establish inter-rater reliability, an initial 30% of the raw data were cross-coded, and a consensus of over 90% was achieved. It is important to clarify that this high level of agreement was reached through an iterative peer-debriefing process, where any initial discrepancies in code assignment were resolved through constant comparison and negotiation until a unified coding scheme was finalized. Furthermore, to enhance the trustworthiness and credibility of the findings, member checking was performed by sharing preliminary thematic maps with a subset of participants (*n* = 5) to verify the accuracy of the interpretations. The researcher also maintained an audit trail of all analytical sessions and the aforementioned peer-debriefing process to ensure the consistency of adaptive behaviors across different contexts, adhering to the standards of trustworthiness in qualitative inquiry (Lincoln & Guba, 1985). The representative reflective journal sample and the corresponding hierarchical coding analysis are provided in Figure S1 and Table S1 of the Supplementary Materials.

## 3. Results

The analysis revealed that pre-service physical education teachers (PSTs) do not simply collapse in the face of environmental constraints; instead, they construct a practical compass through five dynamic coordinates (Table 1). These results demonstrate a transformative process where Deficits are converted into Professional Behavioral Assets through adaptive bricolage. This mechanism ensures the resilience of instructional quality by enabling educators to maintain the continuity of instruction even in unpredictable landscapes.

### 3.1. Coordinate 1: Facing Constraints—The Stalled Map and the Wall of Reality

The first step in creating a compass began with the confirmation of deficit and Ontological Bewilderment (a state of profound professional disorientation), acknowledging that the theoretical Map (the initial lesson plan) was no longer valid. For PSTs, the lesson plan was a sacred document of technical rationality, but the field proved to be an uncontrollable swampy lowland that triggered significant instructional transition challenges.

#### 3.1.1. Epistemic Void

PSTs encountered what we define as the Epistemic Void—a critical gap where abstract pedagogical models learned at university proved insufficient for the non-linear reality of the classroom. For them, the textbook was not a kind guide but an unkind summary with the context removed. PST 16 described the moment of encountering this void as follows:

When I opened the textbook, the content on Tchoukball was only half a page. The rule explanation was ambiguous, and I thought I couldn’t possibly fill a 45 min class with this. I felt a cold sweat running down my back, realizing that the ‘living knowledge’ I had to teach the children was not there. The ‘precise map’ I had relied on for years was hollow.

(PST 16)

#### 3.1.2. Physical Blockade

While the epistemic void concerned content, the Physical Blockade was a problem of environment. The ideal class scenarios were shattered against the physical walls of reality. PST 11 (Group A) vividly described the moment their prepared Map became a discarded draft:

The school weight room was under construction and unusable, and the playground didn’t even have lines drawn, let alone rugby goalposts. The lesson plan I stayed up all night writing was perfect, but there was no stage to put on that play. I had no choice but to stand blankly on the playground like an actor without a stage. My map was useless here.

(PST 11)

This “Ontological Bewilderment” signifies more than a mere lack of preparation; it represents a fundamental crisis of professional identity. The PSTs’ reliance on a “rigid map” acts as a defensive mechanism against the inherent messiness of the classroom. When this map fails, the resulting “Epistemic Void” becomes a transformative space where the PST must stop being a passive consumer of curriculum and start being an active constructor of pedagogical meaning.

### 3.2. Coordinate 2: Resource Mining—Hybrid Bricolage

Having confirmed the absence of official resources, PSTs initiated Resource Mining. This search for survival was a proactive behavioral achievement of agency, effectively transforming environmental deficits into professional pedagogical assets through the creative recombination of tools at hand.

#### 3.2.1. Digital Mining: Algorithmic Solutions

Group A actively utilized generative AI to fill the void. In this context, digital tools functioned as an essential scaffold to navigate the cognitive load of instructional redesign (Zapata-Rivera et al., 2024; Cooper, 2023). PST 1 explained how AI served as a virtual mentor:

I asked Perplexity (AI) for ‘step-by-step drills for badminton beginners.’ The AI even suggested ‘error types’ and ‘correction methods’ that the textbook didn’t tell me. AI gave me reassurance like having a senior teacher with 20 years of experience by my side. Technology became my asset in the face of deficit.

(PST 1)

#### 3.2.2. Cultural Mining: Emotional Solutions

Conversely, Group B focused on mining cultural resources to move students’ minds and hearts. They utilized popular culture as a pedagogical asset to sustain student engagement when technical instructions failed. PST 22 shared:

I was at a loss trying to explain the volleyball spike in words. So, I clipped the spike scene from the animation ‘Haikyu!!’ that students are crazy about. I needed the image of ‘I want to be like that’ which would make children’s hearts beat, rather than just technical precision. Bricolage was my way of finding the ‘heart’ of the sport.

(PST 22)

Resource Mining in this context is an act of “Pedagogical Hacking.” By integrating generative AI with emotional cultural codes (such as animation), PSTs are not just filling a gap; they are creating a hybrid instructional reality. This behavioral achievement demonstrates that teacher agency in the 21st century is increasingly defined by the ability to navigate and synthesize disparate information ecosystems to sustain pedagogical momentum when official resources are absent.

### 3.3. Coordinate 3: Contextual Engineering—Didactic Transposition

Rough materials mined from outside required processing through Didactic Transposition to fit the specific level and context of the school environment. This structural hacking signifies a move away from linear execution toward a more situational, adaptive pedagogical response.

#### 3.3.1. Environmental Hacking: Redesigning the System

Group A attempted Environmental Hacking, a structural approach to redesigning learning tools and flow. PST 2 described her engineering approach to cloning her agency to ensure instruction continuity:

There wasn’t enough time to explain movements one by one. So, I made action cards and placed them all over the court. This had the effect of cloning me and putting me in several places. I became an engineer designing an environment where students could learn on their own, not just a teacher delivering instructions.

(PST 2)

#### 3.3.2. Meaning Translation: Connection Through Metaphor

Group B focused on Meaning Translation, bridging the gap between abstract biomechanical principles and the students’ lived experience through somatic metaphors. PST 28 explained:

Technical instructions like ‘extend your elbows’ didn’t reach the children. Then suddenly, ‘Princess Bow’ came to mind. As soon as I shouted, ‘Now, bow gracefully like a princess at a ball!’, the children’s stiff shoulders relaxed and their movements were corrected as if by magic. It was the moment my language logged into the children’s bodies.

(PST 28)

Contextual Engineering marks the transition from “Technical Rationality” to “Reflexive Practice.” When Group A “hacks” the environment with action cards, they are essentially decentralizing teacher authority to ensure learning continuity. Similarly, Group B’s use of somatic metaphors represents a “Linguistic Bridge” that translates abstract biomechanical principles into the lived experiences of students, thereby reducing the “Physical Blockade” through semiotic adaptation rather than structural change.

### 3.4. Coordinate 4: Simulation—Controlling Uncertainty

PSTs threw their written plans into virtual situations to manage the inherent messiness of the field. This stage represents the behavioral achievement of agency through flexible modification and virtual rehearsal.

#### 3.4.1. De-Scripting: Securing Improvisation

Group A realized through simulation that sentence-type scripts were cognitively restrictive and chose a De-scripting strategy to enhance behavioral flexibility. PST 1 described this behavioral transition from a rigid map to a sustainable, flexible compass:

At first, I wrote the script without missing a word, but in simulation, I got tongue-tied. So, I threw it away and went in with only keywords. Leaving blanks actually gave me the room to speak according to the situation and allowed for more eye contact. It was the transition from a map to a compass.

(PST 1)

#### 3.4.2. Virtual Rehearsal: Somatic Tuning

Group B used the camera to check their physical habits. Through this process, PSTs cultivated an “individual eye”—a unique human observation skill that detects subtle student cues and environmental energy, which automated systems and AI cannot replicate. PST 27 reflected:

Watching the video back, I painfully felt how verbose my explanation was and how unstable my gaze was. Without this process of ‘awkward acting’ rehearsal, I wouldn’t have been able to remove unnecessary clutter from the class and see my own teaching objectively.

(PST 27)

The act of “De-scripting” and “Virtual Rehearsal” represents a courageous behavioral shift. It reveals that professional expertise is not found in the fluency of a pre-written script, but in the “Silence and Blanks” that allow for genuine interaction. By intentionally leaving room for improvisation, PSTs move away from being “Instructional Machines” toward becoming “Responsive Educators” who can read the atmospheric energy of the gym and adjust their own embodied presence accordingly.

### 3.5. Coordinate 5: Reflective Participation—Securing Stereoscopic Vision

The completion of the bricolage compass was the acquisition of Reflective Vision, viewing the class stereoscopically. This ultimate professional asset allows for long-term professional resilience by integrating objective observation and subjective participation.

#### 3.5.1. Observer’s Lens: Detecting Structural Defects

Group A focused on discovering structural and safety defects from a third-party perspective. This shift from doing to seeing allowed them to detect hidden risks that traditional technical rationality often overlooks. PST 15 noted:

I didn’t know when I was teaching, but watching a friend’s class from the outside, I saw safety blind spots in the racket swing radius. Once I had the observer’s eye, I finally started to see the blind spots in my own class. This stereoscopic vision is the ultimate asset I gained from the deficit.

(PST 15)

#### 3.5.2. Participatory Body: Promoting Dynamics

Group B discovered that class became smoother when the teacher entered as an internal participant. This Participatory Body transformed the teacher’s own physical energy into a sustainable teaching resource. PST 12 described the change:

The moment the teacher stopped being stiff and standing in the back and entered as a ‘participant-referee’ or commentator, the children’s eyes changed and the atmosphere of the class became hot. My body became a resource for the class.

(PST 12)

The acquisition of “Stereoscopic Vision” is the final stage of the bricolage journey. By oscillating between the “Observer’s Lens” and the “Participatory Body,” PSTs overcome the blind spots inherent in traditional instructional design. This dual-track reflection ensures that the teacher is both a designer of the environment and a co-creator of the learning experience, solidifying the resilience of the PE ecology and ensuring that the teacher’s own embodied presence remains the most sustainable resource in the face of systemic deficits.

## 4. Discussion

The findings of this study offer a critical departure from traditional, linear models of instructional design. By tracing the journey of secondary PSTs as they navigate the “swampy lowlands” (Schön, 2017) of the classroom, this research suggests that long-term pedagogical continuity does not depend on a pre-set Map but on the behavioral resilience and adaptive Compass of the teacher’s agency. As suggested by Priestley et al. (2015), agency is not a property of the person but an emergent phenomenon through ecological interaction. This shift is essential for ensuring instructional durability in an era where environmental and systemic constraints are the norm.

### 4.1. From Linear Planning to Adaptive Behavioral Mechanisms: The Dialectics of Map and Compass

First, this study reconceptualizes instructional design from a linear process to a non-linear behavioral adaptation mechanism. Traditional PETE has defined ID as a technical procedure of creating error-free plans based on technical rationality (Tyler, 2013). However, actual PE classes are complex environments where uncontrollable variables abound (Casey, 2014). It is important to note that the ‘Compass’ does not replace the ‘Map’; rather, it represents the professional capacity to internalize the Map’s theoretical essence into situational action.

The ability to navigate this complexity is a core component of adaptive expertise, which differentiates resilient educators from those who rigidly adhere to a failed plan (Carbonell et al., 2014). The psychological shock (Ontological Bewilderment) experienced by PSTs is not an indicator of professional deficit but a “necessary cognitive dissonance” that triggers the transition from a ‘curriculum consumer’ to a ‘pedagogical architect.’ This demonstrates that agency is a dynamic achievement of professional behavior rather than a fixed capacity.

### 4.2. Embodied Agency: The Teacher as a Dynamic Behavioral Asset

Second, the typology of bricolage strategies elucidated the behavioral trajectories of PSTs. By categorizing strategies into Analytical/Log-centric (Group A) and Narrative/Somatic (Group B), this study reveals that teacher agency is a multifaceted behavioral achievement shaped by individual psychological dispositions. While Group A focused on systematic resource procurement and structural redesign, Group B prioritized affective engagement and embodied pedagogical performance to maintain instructional momentum.

Crucially, this Embodied Agency highlights the ‘Individual Eye’—the human capacity for somatic empathy and situational awareness that automated pedagogical tools cannot replicate. These somatic metaphors (Group B) act as behavioral assets that ensure learning continuity when technical instructions fail. This study demonstrates that the teacher’s embodied presence and psychological resilience function as critical instruments of practice (Barker et al., 2015), transforming the teacher into a sustainable and resilient resource within the classroom ecology.

### 4.3. Reinterpreting ‘Deficit’ as a Psychological Catalyst: The ‘Safe Deficit’ Device

Third, Deficit was reinterpreted as an Ecological Catalyst for behavioral growth. While previous scholars like O’Sullivan (2006) have expressed concern over instructional transition challenges leading to burnout, this study found that when a deficit is deliberately managed within a ‘Safe’ and supportive pedagogical scaffold, it functions as a powerful trigger awakening latent agency. By reframing the potential ‘reality shock’ into a ‘resilience trigger’, this research introduces the Safe Deficit paradigm not as a glorification of lack but as a strategic pedagogical device. To prevent the risk of chronic burnout, the ‘Safe’ in ‘Safe Deficit’ must be carefully calibrated by PETE programs to ensure that the challenge stimulates growth without overwhelming the PST’s psychological durability.

This aligns with the ecological agency perspective (Priestley et al., 2015), which posits that agency is achieved in interaction with the environment. Encountering disorienting dilemmas or managed deficits is a prerequisite for transformative learning and the development of a resilient professional identity (Mezirow, 1991; Beltman et al., 2011). By embracing the Safe Deficit paradigm, teacher education can foster proactive behavioral resilience that goes beyond mere survival to thriving in pedagogical uncertainty (Gu & Day, 2007). PETE should actively utilize Safe Deficit as a strategic psychological tool to stimulate problem-solving skills, ensuring that PSTs enter the profession with a durable set of adaptive behaviors.

This paradigm shift suggests that professional expertise in secondary education is not an accumulation of static knowledge but a dynamic behavioral plasticity developed through situational negotiation. By experiencing ‘Safe Deficits,’ PSTs do not merely learn to cope; they undergo a behavioral transformation that fosters long-term psychological durability. This highlights the importance of incorporating unpredictable environmental variables into teacher education to build a more resilient and adaptive teaching force capable of sustaining educational quality amidst global uncertainties.

## 5. Conclusions

This study demonstrated that the instructional design journey of secondary pre-service physical education teachers (PSTs) begins with the loss of a rigid map and is completed with the discovery of an adaptive behavioral compass. Rather than acting as technical engineers in a sterile environment, the participants functioned as bricoleurs who transformed instructional transition challenges into creative behavioral opportunities. The findings shift the focus from the perfection of instructional products to the resilience of behavioral processes, providing a new roadmap for fostering professional agency in teacher education.

### 5.1. Educational Implications for Resilient Teacher Agency

- From Smooth Planning to Embracing Rough Reality: Current teacher education curricula are often overly focused on lesson demonstrations in sterile and ideal environments, which leaves PSTs psychologically vulnerable to the unpredictable nature of real-world classrooms. To foster true professional resilience, teacher education must intentionally introduce “Safe Deficit” simulations that trigger adaptive behavioral responses while carefully calibrating the degree of challenge to prevent psychological overload or burnout. By limiting resources or imposing unexpected classroom variables within a supportive “Safe Zone”, PSTs can train their behavioral plasticity. This pedagogy of uncertainty does not discard the theoretical “Map” but rather uses it as a foundational springboard to develop a responsive “Compass”, ensuring instructional continuity through internal adaptive capacity rather than external abundance.
- Cultivating Signature Pedagogy through Disposition-based Bricolage: Teacher education should move away from instilling a uniform “good class” model that stifles individual teacher agency. Instead, it should be a process of behavioral individuation, helping PSTs discover their own Signature Pedagogy based on their psychological dispositions. As evidenced by the distinct strategies of Group A (Structural) and Group B (Embodied), teacher agency is a multifaceted behavioral achievement. Providing differentiated feedback that respects these individual psychological traits and somatic sensitivities will lead to a more resilient and diverse teaching force, capable of addressing student needs through a wide spectrum of adaptive teaching behaviors.

### 5.2. Limitations and Future Directions

While this study provided deep insights within the context of micro-teaching, actual school environments possess far more complex, multi-layered dynamics. Future research should conduct longitudinal studies to track the behavioral evolution of these bricolage competencies amidst the bureaucratic cultures and systemic administrative constraints of actual schools. Furthermore, investigating the longitudinal impact of systematically designed “Safe Deficit” training on actual teacher retention rates and psychological well-being would provide valuable empirical evidence for systemic policy changes in teacher induction.

In conclusion, this research suggests that the future of teacher education lies in fostering “bricoleur-teachers”—resilient educators who can navigate pedagogical crises with proactive agency (Priestley et al., 2015), thereby ensuring the long-term quality and psychological durability of education in an unpredictable world.

## Figures and Tables

**Table 1:** Matrix of Adaptive Instructional Design Bricolage Strategies.

Coordinates	Challenges	Group A (Analytical/Log-Centric)	Group B (Narrative/Somatic)
1. Facing Constraints	Void & Blockade	Epistemic Diagnosis	Physical Sensing
2. Resource Mining	Resource Deficit	Digital Mining (AI)	Cultural & Emotional Mining
3. Contextual Engineering	Implementation Gap	Environmental Hacking	Meaning Translation (Metaphor)
4. Simulation	Uncertainty	De-scripting	Virtual Rehearsal
5. Reflective Participation	Blind Spots	Observer’s Lens	Participatory Body

Note: The categorization of participants into Group A (Analytical/Log-centric) and Group B (Narrative/Somatic) was not a pre-determined experimental condition or a pre-set grouping. Instead, it emerged as an inductive finding from the thematic analysis, representing two distinct psychological and behavioral response trajectories to instructional deficits within the PE ecology.

## Data Availability

The data presented in this study are available on request from the corresponding author. The data are not publicly available due to privacy and ethical restrictions regarding the identity of pre-service teachers.

## Supplementary Materials

The following supporting information can be downloaded at: https://www.mdpi.com/article/10.3390/bs16040515/s1, Figure S1: Sample of a PST’s (PST 3) reflective journal; Table S1: Detailed Analysis of the Representative Case (PST 3).

## Abbreviations

The following abbreviations are used in this manuscript:

PST	Pre-service Teacher
PETE	Physical Education Teacher Education
PE	Physical Education
ID	Instructional Design
AI	Artificial Intelligence
IRB	Institutional Review Board
