# Supplementary material for: Turning Constraints into Adaptive Behavior: Secondary Pre-Service Teachers’ Bricolage and Agency in Physical Education
Source: Behav Sci (Basel). 2026 Mar 29;16(4):515. doi: 10.3390/bs16040515 (PMC13113363; doi:10.3390/bs16040515)
Supplement: Supplementary file 1 [file behavsci-16-00515-s001.zip › behavsci-4159572-supplementary.pdf]

Supplementary Data: Representative Reflective Journal and Analysis

Figure S1. Sample of a PST's (PST 3) reflective journal.

(Note: To ensure anonymity, the participant's real name and student ID have been redacted. The text highlights the adaptive use of AI and instructional strategy modifications.)

25-2 Research and Methods in Physical Education Teaching Materials 2025.12.14.

Reflections on Research and Plactice of PE Teaching Materials

1. Reflections on the Second Micro-teaching Session

Unit/Topic: Sports_Badminton

In this session, I applied the Sport Education Model. The most significant change was omitting the instructional handouts. I realized that for effective strategy delivery, a single, powerful point is more effective than a verbose guide. I focused on the keyword "Finding & Blocking Empty Spaces." Also, during the video analysis, I noticed that he that students might focus more on the movement of the racket than the teacher's voice. I learned the importance of refined physical movements and minimizing distractions. The professor's feedback likened my delivery to a "star lecturer," which gave me great confidence.

2. Reviewing the Process of Research and Practice

I have summarized the preparation process experienced while preparing for several classes in this course.

Preparation Steps:

1. Check page numbers in textbooks for the physical activity:
   - PST's Perspective: Search for PDF textbooks using AI "Perplexity".
2. Select one from multiple textbooks (standardized or school-specific).
3. Check achievement standards and the specific lesson sequence.
4. Construct unit plans based on standards and models (e.g., Sport Education Model).
5. Draft detailed lesson plans: Minimize management time by using PPT or diverse teaching aids. Check everything from the student's perspective.
6. Practice through micro-teaching rehearsals.

3. Course Conclusion / Final Thoughts

This course, taken just before the teaching practicum, provided invaluable practical knowledge. Through micro-teaching and direct feedback, I identified specific areas for improvement in my own teaching. The professor's detailed guidance has significantly boosted my professional confidence.

Table S1. Detailed Analysis of the Representative Case (PST 3).

Coordinate	Specific Action & Reflection	Related Result Section
Coordinate 2: Resource Mining	Utilized the AI tool 'Perplexity' to overcome the lack of physical resources and locate diverse pedagogical materials.	3.2.1. Digital Mining
Coordinate 3: Contextual Engineering	Simplified complex instructions into a single strategic keyword: "Finding the Empty Space" for better student understanding.	3.3.2. Meaning Translation
Coordinate 4:	Based on instructional simulation, the teacher	3.4.1. De-

Coordinate	Specific Action & Reflection	Related Result Section
Simulation	boldly omitted written handouts to prioritize real-time eye contact and engagement.	scripting
Coordinate 5: Reflective Participation	Shifted from a static instructor to an active "participant-referee," using the teacher's embodied presence as a teaching asset.	3.5.2. Participatory Body
